# Combined morphological and functional liver MRI using spin-lattice relaxation in the rotating frame (T1ρ) in conjunction with Gadoxetic Acid-enhanced MRI

**DOI:** 10.1038/s41598-018-37689-y

**Published:** 2019-02-14

**Authors:** Jonas D. Stief, Moritz Haase, Lutz Lüdemann, Dorothea Theilig, Moritz Schmelzle, Bernd Hamm, Timm Denecke, Dominik Geisel

**Affiliations:** 1 Department of Diagnostic and Interventional Radiology, Charité Campus Virchow-Klinikum, Augustenburger Platz 1, 13353 Berlin, Germany; 2 Department of Medical Physics, Essen University Hospital, Essen, Germany; 3 Department of General, Visceral and Transplantation Surgery, Charité Campus Virchow-Klinikum, Berlin, Germany

## Abstract

Noninvasive early detection of liver cirrhosis and fibrosis is essential for management and therapy. The aim was to investigated whether a combination of the functional parameter relative enhancement (RE) on Gadoxetic Acid magnetic resonance imaging (Gd-EOB-DTPA-enhanced MRI) and the fibrosis parameter T1ρ distinguishes cirrhosis and healthy liver. We analyzed patients with Gd-EOB-DTPA-enhanced MRI and T1ρ mapping. Signal intensity was measured before and after contrast; RE was calculated. T1ρ was measured with circular regions of interest (T1ρ-cROI). A quotient of RE and T1ρ-cROI was calculated: the fibrosis function quotient (FFQ). Cirrhosis was evaluated based on morphology and secondary changes. 213 datasets were included. The difference between cirrhotic and noncirrhotic liver was 51.11 ms vs. 47.56 ms for T1ρ-cROI (p < 0.001), 0.59 vs. 0.70 for RE (p < 0.001), and 89.53 vs. 70.83 for FFQ (p < 0.001). T1ρ-cROI correlated with RE, r = −0.14 (p < 0.05). RE had an AUC of 0.73. The largest AUC had the FFQ with 0.79. The best cutoff value was 48.34 ms for T1ρ-cROI, 0.70 for RE and 78.59 ms for FFQ. In conclusion T1ρ and RE can distinguish between cirrhotic and noncirrhotic liver. The FFQ, which is the combination of the two, improves diagnostic performance.

## Introduction

There is a growing worldwide epidemic of chronic liver disease^1^. Malnutrition, chronic viral hepatitis, and chronic alcohol abuse result in a significant increase in alcoholic and non-alcoholic fatty liver disease and steatohepatitis. These changes progress to fibrotic remodeling of the liver parenchyma, which can ultimately lead to cirrhosis. Both cirrhosis and its pre-stages are associated with an increased occurrence of hepatocellular carcinoma^2^. The final stage of liver cirrhosis is an irreversible condition, which makes it necessary to detect the fibrotic pre-stages at an early point in order to be able to treat them immediately^3^. Liver biopsy is the gold standard to detect and quantify liver fibrosis and cirrhosis. However, biopsy is an invasive procedure with serious complications^4,5^ and is limited by potential sampling errors^6^. It is therefore not useful for regular follow-up of high-risk patients. Consequently, a tool is needed that offers a noninvasive and easy way to detect liver fibrosis at an early stage and quantify its progression^7,8^. Many patients undergo regular follow-up magnetic resonance imaging (MRI) of the liver after oncological therapy. These include patients who have been treated by interventional radiological procedures of the liver such as selective internal radiotherapy (SIRT), transarterial chemoembolization (TACE), CT-guided high-dose-rate brachytherapy (CT-HDRBT), and radiofrequency ablation (RFA). These patients have an increased risk of parenchymal and functional hepatic changes from their underlying disease or their interventions. Therefore, it is important to monitor hepatic parenchyma to ensure that therapies are initiated in time to maintain function or to limit therapies should there be an increased risk of liver insufficiency.

There are several ways to quantify parenchymal and functional changes of the liver. Several methods to analyze the liver structure have been investigated. Currently applied methods include shear-wave elastography (SWE), MR elastography (MRE), T1-mapping, and T1rho imaging (T1ρ). T1rho relaxation time or spin lattice relaxation time in the rotating frame is the transverse magnetization decay during a continuous radiofrequency pulse (RF). The pulse is applied along the transverse decay. It is assumed that T1ρ detects slow-frequency motion of macromolecules. Many studies have shown that T1ρ increases with the degree of liver fibrosis and can distinguish the different stages. This applies to both 1.5 Tesla MRI and 3.0 Tesla MRI^9–15^. Investigations in animal models show that T1ρ decreases again once the factor causing liver fibrosis has been eliminated^16,17^. These results indicate that T1ρ also has the potential to be used for therapy monitoring. In musculoskeletal imaging, it has already been shown that the combination of T1ρ and contrast agent can provide additional information^18^. More studies, especially in a clinical setting, are needed to establish T1ρ as a reliable biomarker for differentiating between fibrosis degrees and liver cirrhosis stages.

Besides using MRI for assessing parenchymal changes, it is also possible to image functional changes. One method is gadolinium-ethoxybenzyl-diethylenetriamine penta-acetic acid (Gd-EOB-DTPA) enhanced MRI. Gd-EOB-DTPA is a specific contrast agent that is taken up by hepatocytes and eliminated biliary. Uptake of this contrast agent into liver cells can be estimated by measuring relative enhancement (RE). It has been shown that hepatocellular uptake of Gd-EOB-DTPA correlates with cirrhosis stages and with laboratory parameters of liver function^19–21^. In addition, it has been shown that RE correlates with retransplantation-free survival after liver transplantation^22^.

The aim of this retrospective study is (1) to evaluate whether there is a correlation between RE and T1ρ. (2) To investigated whether combination of the two imaging tests allows reliable diagnosis or exclusion of liver cirrhosis. For this reason, we are introducing the fibrosis function quotient (FFQ). The FFQ is the quotient of the T1ρ value and RE. This value combines the morphological and functional changes of the liver. T1ρ represents the morphological changes and RE the functional changes. Since previous studies have shown that T1ρ increases as a result of fibrosis while RE decreases, it was decided to form a quotient^9,20^. Thus, increasing FFQ values indicate an increasing impairment of the liver. (3) to determine whether T1ρ is useful in patients after interventional oncological procedures.

## Materials and Methods

### Inclusion and exclusion criteria

A retrospective analysis of patients who underwent MRI of the liver with Gd-EOB-DTPA as contrast agent and T1ρ mapping in our department from May 2016 through March 2017. This study was approved by the IRB. The ethic committee waived informed consent requirements for this retrospective study (Ethikkommission der Charité – Universitätsmedizin Berlin, Charitéplatz 1, 10117 Berlin, Germany). Exclusion criteria were as follows: A compromised measurement due to increased specific absorption rate (SAR), extensive right liver resection, severe artifacts, hemochromatosis, diffuse metastasis of the whole liver, and presence of very large tumors without enough remaining intact tissue for measurement.

### MRI

Patients underwent MRI in a 1.5 Tesla Siemens Magnetom Avanto MRI scanner (Siemens Healthcare, Erlangen, Germany) using the one channel body coil and 8-channel surface coils to transmit and receive signal, respectively.

#### T1ρ

T1ρ-weighted images were acquired before contrast agent administration. The spin lock sequence was provided by the Center for Magnetic Resonance & Optical Imaging of the Perelman School of Medicine, University of Pennsylvania^23^. T1ρ-prepared magnetization was imaged with a single-slice 2D Fast Low-Angle Shot (FLASH) readout with the following parameters: TR/TE 5.1 ms/2.4 ms, flip angle 10° (α), FOV 30 cm × 30 cm, slice thickness 10 mm, matrix size 128 × 128, averages 1, and scan time for each spin lock time approximately 2.5 sec. T1ρ-weighted contrast was yielded by a non-spatially selective spinlock (SL) preparation consisting of a 90° tip down pulse, SL pulse with frequency fixed at 500 Hz, 180°, opposite phase SL, 90° tip up pulse, followed by a crusher gradient^23^. A series of six T1ρ-weighted images was acquired on each slice with the following spin lock times (T_SL_): 0, 10, 20, 30, 40, and 50 ms. A total of two slices were acquired: One 3 cm cranial and one 3 cm caudal of the hilus.

The image datasets were processed offline with OsiriX lite 7.0.3 using the T2 exponential regression plugin. The T1ρ signal decays exponentially with the spin lock time, see eq. 1 in^23^, as by echo time, see eqs 1 and 2 at Regatte *et al*.^24^. TE was manually replaced in the plugin by the actual spin lock time thus calculating T1ρ instead of T2. TE values were manually replaced by T_SL_ values. A series of at least five T1ρ-weighted images was used to generate T1ρ maps by fitting every pixel expression to calculate the T1ρ value using the linear least-squares method:

$$ln(\frac{S({T}_{SL})}{{S}_{0}})=-\,\frac{{T}_{SL}}{{T}_{1\rho }}+C$$

where S(T_SL_) is the measured signal intensity of the image at a particular T_SL_, S_0_ is the signal intensity at T_SL_ = 0, and C is an intercept. The opposite phase of the T1ρ spin locking pulses and 180 degree pulse reduced the effects of B1 RF inhomogeneity as proposed by Weitian Chen^25^.

#### Gd-EOB-DTPA-enhanced MRI

Images were acquired before and 20 min after manual bolus injection of 0.1 ml/kg body weight of Gd-EOB-DTPA (Primovist, Bayer, Berlin, Germany). A volume-interpolated breath-hold examination sequence (VIBE) in an axial plane with a TR of 4.26 ms, a TE of 1.93 ms, a flip angle of 10°, a slice thickness of 3 mm, and a matrix size of 256 × 127 was acquired covering the entire liver with 60–72 slices and an adjusted field of view (FOV) of 255–300 × 340–400 mm. A dose of 0.1 ml of Gd-EOB-DTPA (0.25 mmol/ml) per kg body weight was then manually injected into an antecubital vein, followed by a saline flush of 20 ml. After 20 min, in the hepatobiliary phase, the same sequence was acquired again.

### Image analysis

**Figure 1:**
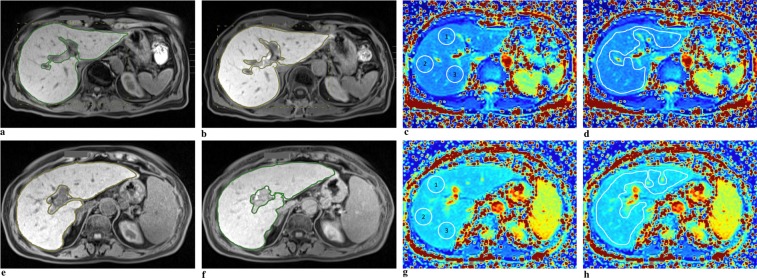
Image analysis. Normal liver parenchyma on image (**a**–**d**). Liver cirrhosis on image (**e**–**h**). On image (**a** and **e**) measurement of signal intensity (SI) pre-contrast. On image (**b** and **f**) measurement of SI post-contrast. On image (**c** and **g**) measurement of T1ρ with 3 regions of interest (ROI) on the right liver lobe (T1ρ circular ROI). On image (**d** and **h**) measurement of T1ρ with one ROI over the whole liver.

To obtain mean T1ρ values, two methods were used (Fig. 1) with the reader blinded to the underlying condition and laboratory data:

1. Three circular regions of interests (ROIs) were manually placed on each slice of the T1ρ map using Visage 7.1.4 (Visage Imaging, Richmond, NSW, Australia). Many publications have already used this method^13,16,17,26–29^. In this project we have kept to the instructions of these publications. One ROI was approx. 2–3 cm in diameter. The ROI was placed on the right liver lobe (RLL) – anteriorly, centrally, and posteriorly on each slice. The left liver lobe (LLL) was not assessed due to frequent pulsation artifacts of the heart. Large vessels were omitted. Tumor and ablation areas were avoided. A distance of approximately 1 cm from the edge of the liver was maintained. A total of 6 ROIs per liver were measured. In some MRI examinations, individual liver anatomy precluded imaging of 2 planes. In these cases, the number of ROIs was reduced accordingly. The mean T1ρ value for circular ROIs was calculated (T1ρ-cROI). The unit for T1ρ-cROI is milliseconds.
2. A single ROI was placed over the entire liver in all 2 slices using Visage 7.1.4. A number of publications have already used this method^12,30^. A distance of approximately 1 cm from the edge of the liver was maintained. Again, the large vessels, tumor, and ablation areas were avoided. The mean T1ρ value for the whole liver was calculated (T1ρ-wl). The unit for T1ρ-wl is milliseconds.

Signal intensity (SI) was measured before and 20 min after contrast agent administration using Visage 7.1.4. One ROI per slice was placed over the whole liver (Fig. 1). Large vessels, tumor and ablation areas were avoided. Subsequently, the average was calculated. RE was calculated according to the following formula:

$$RE=\frac{({{\rm{SI}}}_{20min}\,-\,{{\rm{SI}}}_{{\rm{unenhanced}}})}{{{\rm{SI}}}_{{\rm{unenhanced}}}}$$

Then the Fibrosis Function Quotient was calculated as follows:

$$FFQ=\frac{{\rm{T}}1{\rm{\rho }}\,{\rm{cROI}}}{{\rm{RE}}}$$

### Statistical Analysis

Statistical analysis was performed using SPSS Statistics 24 (IBM, Armonk, NY, USA). Receiver operating characteristic (ROC) curves were created. A positive value is defined as presence of liver cirrhosis. Cutoffs were determined using Youden’s index. Area under the ROC curve, sensitivity, specificity, positive likelihood ratio (PLR), and negative likelihood ratio (NLR) were calculated. Negative and positive predictive values were not calculated because our study population is not representative of the true prevalence of liver cirrhosis. Student’s *t*-test was performed to assess differences in T1ρ values, RE, and FFQ between patients without and with liver cirrhosis. Pearson’s *r* was calculated to analyze correlation between T1ρ and RE. A *P* value of <0.05 was considered statistically significant. All quantitative data are expressed as mean ± standard deviation (SD), unless otherwise indicated.

## Results

**Figure 2:**
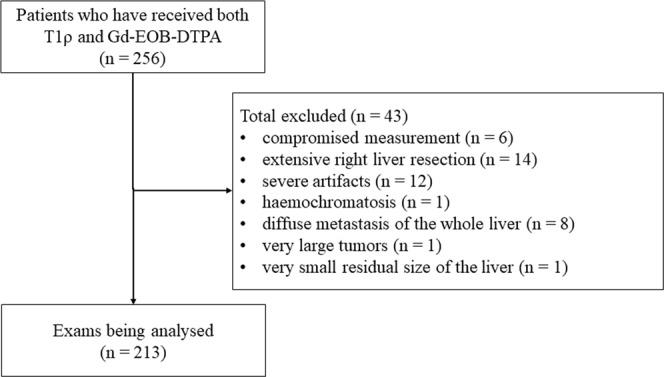
Patient selection and exclusion criteria. Gd-EOB-DTPA (gadolinium-ethoxybenzyl-diethylenetriamine penta-acetic acid).

A total of 256 MRI examinations were screened for inclusion in this retrospective analysis. Forty-three MRI datasets were excluded, leaving 213 datasets for inclusion into our analysis (Fig. 2). The patients had an average age of 65.2 ± 13.9 years (range 23–88). Of the included patients, 129 were female and were 84 male. A total of 150 patients had one or multiple interventional therapies of the liver prior to imaging: 127 CT-HDRBT, 46 TACE, 21 SIRT, and 6 RFA. Some patients had more than one of these interventional treatments. 47 patients had liver cirrhosis, 166 patients had no sign of cirrhosis.

**Figure 3:**
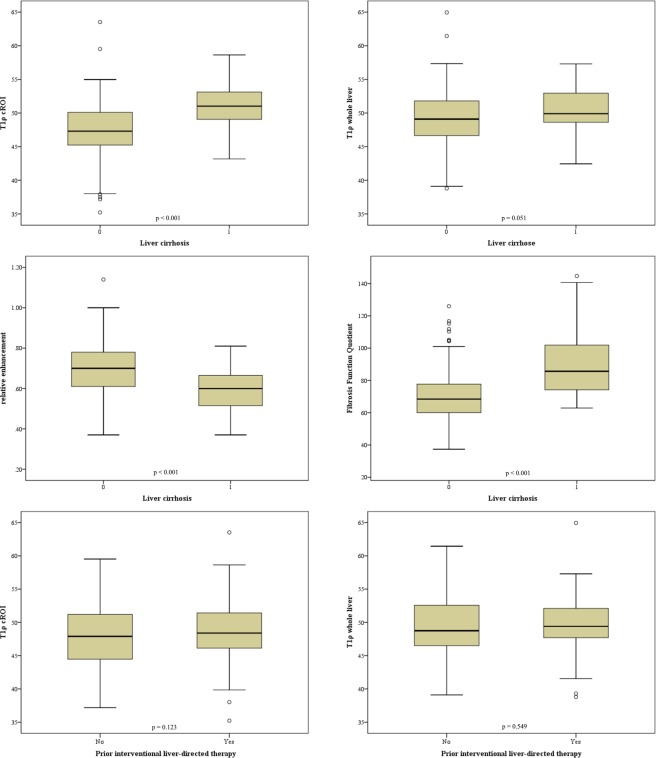
Boxplots for the differentiation between liver cirrhosis and noncirrhotic liver parenchyma for the parameters T1ρ circular region of interest (cROI), T1ρ whole liver, relative enhancement and fibrosis function quotient. And boxplots for the differentiation between liver with and without prior interventional liver-directed therapies (bottom line).

Results of the differentiation between normal liver tissue and cirrhosis are compiled in Table 1 and Fig. 3. Mean T1ρ measured in circular ROIs was 47.56 ms ± 4.17 ms in noncirrhotic liver versus 51.11 ms ± 3.45 ms in cirrhotic liver; the difference was significant (p < 0.001). T1ρ values measured over the whole liver were 49.14 ms ± 4.21 ms and 50.44 ms ± 3.16 ms, respectively (p > 0.05). With both methods, T1ρ was not significantly different between patients with and without prior radiologic interventions with a mean T1ρ value of 49.54 ms ± 3.82 ms vs. 49.15 ms ± 4.51 ms (p = 0.55) and 48.63 ms ± 4.12 ms vs. 47.64 ms ± 4.48 ms (p = 0.12) (Fig. 3). RE was significantly different between patients with and without cirrhosis (0.59 ± 0.11 vs. 0.70 ± 0.13; p < 0.001). The FFQ of patients with cirrhosis was significantly different from that of patients without cirrhosis (89.53 ± 19.84 vs. 70.83 ± 15.56; p < 0.001). T1ρ-cROI had a weak but significant correlation with RE, r = −0.14 (p < 0.05). T1ρ-wl, however, did not show any significant correlation with RE (r = 0.09; p = 0.25). There was no significant correlation between age and T1ρ-cROI and T1ρ-wl. But there was a significant correlation between age and RE (r = −0.21) and between age and FFQ (r = 0.18). The results for gender were similar: there was no significant correlation between gender and T1ρ, but a significant correlation between gender and RE (r = −0.36) with women having higher RE. There was also a weak but significant correlation between FFQ and gender (r = 0.28) with women having lower values.

**Table 1:** Results in the differentiation between liver cirrhosis and healthy liver. Results for the measurement of T1ρ circular region of interest (cROI), T1ρ whole liver (wl), relative enhancement (RE) and fibrosis function quotient (FFQ) in the differentiation between liver cirrhosis and noncirrhotic liver parenchyma. And T1ρ-values for the differentiation between liver with prior interventional radiology and liver without prior interventional radiology.

	Cirrhosis	Mean	Standard Deviation	p-value
T1ρ cROI	Yes (n = 47)	51.11	3.45	p < 0.001
	No (n = 166)	47.56	4.17	
T1ρ wl	Yes	50.44	3.16	p = 0.051
	No	49.14	4.21	
RE	Yes	0.59	0.11	p < 0.001
	No	0.70	0.13	
FFQ	Yes	89.53	19.84	p < 0.001
	No	70.83	15.56	
	Interventional radiology			
T1ρ cROI	Yes (n = 150)	48.64	4.17	p = 0.123
	No (n = 63)	47.64	4.48	
T1ρ wl	Yes	49.54	3.82	p = 0.549
	No	49.15	4.51

**Figure 4:**
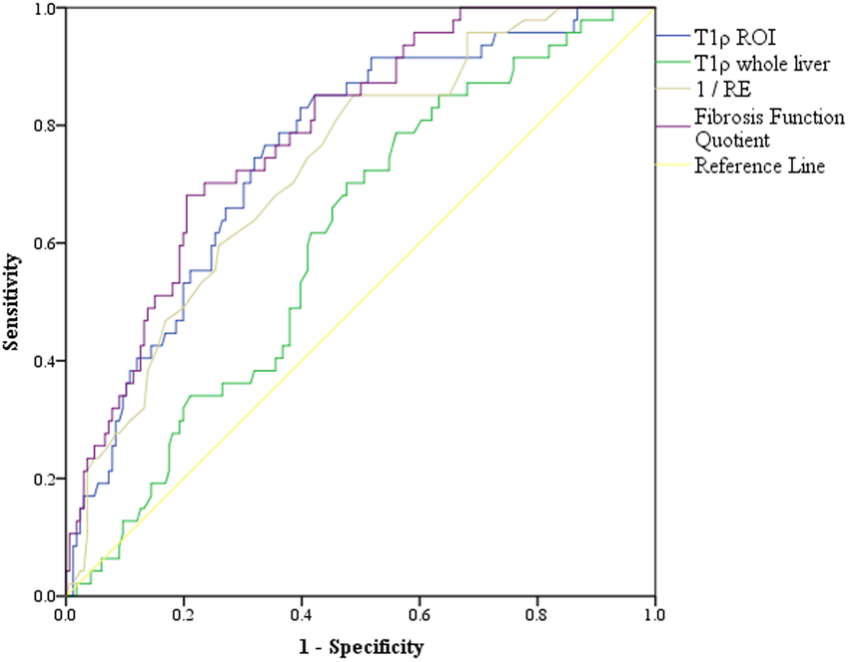
ROC-curves for the differentiation between liver cirrhosis and noncirrhotic liver parenchyma of T1ρ circular region of interest (cROI), T1ρ whole liver, relative enhancement and fibrosis function quotient.

ROC curves were created to evaluate the diagnostic performance of the diagnostic parameters regarding the distinction between cirrhotic and noncirrotic livers (Fig. 4 and Table 2). T1ρ and FFQ are above the diagonal reference line because higher values imply higher rates of cirrhosis, while RE would be below the reference line due to its negative correlation with cirrhosis. For an easy comparability in the ROC diagram we have formed the reciprocal of RE (1/RE). T1ρ-cROI showed a better AUC than T1ρ-wl (AUC = 0.76 vs. AUC = 0.61). RE had an AUC of 0.73. The largest AUC was found for FFQ with 0.79. The best cutoff according to Yourden’s index was 48.34 ms for T1ρ-cROI, 0.70 for RE, and 78.59 ms for FFQ. The corresponding sensitivity and specificity were 83.0% and 60.2% for T1ρ-cROI, 85.1% and 51.2% for RE, and 70.2% and 76.5% for FFQ.

**Table 2:** Diagnostic performance. Diagnostic performance of T1ρ circular region of interest (cROI), T1ρ whole liver (wl), relative enhancement and fibrosis function quotient in the differentiation between liver cirrhosis and noncirrhotic liver parenchyma.

Statistics	T1ρ (cROI)	T1ρ (wl)	Relative enhancement	Fibrotic function quotient
Area und the ROC curve	0.76	0.61	0.73	0.79
Cut off value	48.34	49.18	0.70	78.59
Sensitivity (%)	83.0	70.2	85.1	70.2
Specificity (%)	60.2	52.4	51.2	76.5
Positive likelihood ratio	2.09	1.48	1.84	2.99
Negative likelihood ratio	0.28	0.57	0.29	0.39

## Discussion

In this retrospective analysis of a heterogenous group of patients who underwent MRI of the liver, we assessed T1ρ and relative enhancement after Gd-EOB-DTPA administration for possible correlation in an attempt to improve the distinction between liver cirrhosis and normal liver tissue. We found a weak but significant negative correlation between T1ρ and RE. In terms of diagnostic performance, we found that both parameters had a fair AUC for distinguishing between cirrhotic and noncirrhotic liver. Both parameters had high sensitivity but lower specificity. To compensate for this limitation, we investigated a new parameter - the FFQ, which is a combination of both parameters and is defined as T1ρ divided by RE. The FFQ had a larger, but still fair, AUC and specificity than either T1ρ or RE alone. Overall, our results show that FFQ is a suitable parameter to distinguish cirrhotic liver from noncirrhotic liver.

The T1ρ cutoff we identified for differentiation between cirrhosis and healthy liver is higher than found in one earlier study^10^ but in line with the results of two other studies, which report nearly the same cutoffs^30,31^. Most previous studies showed higher AUC and better diagnostic performance for T1ρ. This discrepancy may be attributable to the fact that we investigated a very heterogenous patient population. Some investigators have also demonstrated that a distinction between CHILD-A to CHILD-C cirrhosis stages is possible^10,31^. Another study, however, did not detect any significant differences among these stages^30^. In addition, there is also a study that did not find any significant differences between the stages of fibrosis^27^. Prior radiologic interventions and hepatic diseases may have altered the liver parenchyma in our patients, which probably leads to artificially elevated T1ρ values in patients without cirrhosis. However, we found no significant difference between patients with a history of radiologic interventions and those without. Only one other published study with a retrospective design was performed in a heterogenous patient population^27^. However, that study did not find significant differences between degrees of fibrosis. It may be easier to distinguish between normal liver and cirrhosis than between the different degrees of fibrosis. Additionally, a higher number of cases and a more robust measuring method regarding the acquisition of T1ρ is required. A more robust T1ρ could be achieved by using a MRI with 3 Tesla. In addition, more spinlock times or artifact reduction sequences might lead to better results.

We compared two methods of determining mean T1ρ: one ROI covering the entire liver in two slices vs. three circular ROIs in the right liver on two slices. The method measuring this parameter in circular ROIs was superior in all aspects to the method using one ROI covering the entire liver. It correlated better with RE and had better diagnostic performance. The reason for this might be that vessels or artifacts are included in the whole-liver ROI thus distorting the T1ρ value of the parenchyma. Especially medium-sized vessels are included in the measurement and these distort the result. In contrast, individual ROIs provide more accurate measurements as they can be placed in areas free of vessels and artefacts.

Our study has some limitations. Since our patients were examined as a proof of concept in the setting of regular screening and clinical routine, we analyzed neither laboratory parameters nor histologic findings. For this reason, we were not able to classify cirrhosis histopathologically. This may have had both positive and negative effects on the cutoff values and diagnostic performance we found, although the diagnosis of cirrhosis can be made confidently using a combination of imaging and clinical parameters. In addition, the lack of clinical and histopathologic information means that possible preliminary hepatic diseases were unknown and could affect the result. In future prospective studies a multi-variable regression analysis with clinical and laboratory parameters should be performed to identify possible confounder parameters and further improve diagnostic performance. One limitation concerns the methodology of the study: The subjective aspect of the ROI placement, even with the instructions given in former studies, could make it difficult to reproduce the results. However, some studies have shown that a good interobserver reproducibility exists^26,27,32^. Another limitation of all studies investigating T1ρ is the diversity of sequence protocols and image analysis. This is reflected by the fact that each group has so far identified different cutoffs. Establishment of a standardized T1ρ sequence and tool for image analysis is essential for comparability of data. This is also necessary to establish T1ρ imaging in clinical routine.

## Conclusion

In conclusion, this study demonstrates that there is a correlation between T1ρ and relative enhancement after Gd-EOB-DTPA administration. The FFQ combines the two parameters and improves diagnostic performance in detecting liver cirrhosis. In addition, our results show that T1ρ also works in patients with previous interventional oncological therapies as long as the affected liver areas are not included in the measurement, therefore extending T1ρ imaging to more real-world scenarios.

## Supplementary information

Supplementary Dataset 1

## Data Availability

All data generated during this study are included in the Supplementary Information files. Due to local regulations, the images are not allowed to be published open to the public. But they can be made available from the corresponding author on request.
